# The Vermetidae of the Gulf of Kachchh, western coast of India (Mollusca, Gastropoda)

**DOI:** 10.3897/zookeys.555.5948

**Published:** 2016-01-20

**Authors:** Devanshi MukundRay Joshi, Pradeep C. Mankodi

**Affiliations:** 1 Senior Research Fellow, Gujarat Ecological Education and Research (GEER) Foundation, Indroda Nature Park, Gandhinagar – 382007, Gujarat, India; 2 Head, Department of Zoology, Faculty of Science, Maharaja SayajiRao University of Baroda, Vadodara – 390002, Gujarat, India

**Keywords:** Coral reefs, Vermetidae, Paga Reef, Gulf of Kachchh

## Abstract

Coral reefs are often termed underwater wonderlands due to the presence of an incredible biodiversity including numerous invertebrates and vertebrates. Among the dense population of benthic and bottom-dwelling inhabitants of the reef, many significant species remain hidden or neglected by researchers. One such example is the vermetids, a unique group of marine gastropods. The present study attempts for the first time to assess the density and identify preferred reef substrates in the Gulf of Kachchh, state of Gujarat, on the western coast of India. A total of three species of the family Vermetidae were recorded during the study and their substrate preferences identified.

## Introduction

**Figure 1.**
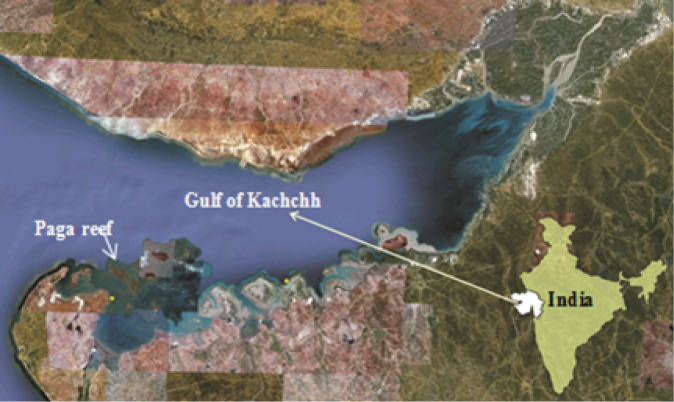
Map showing the study site in the Gulf of Kachchh, Gujarat, west coast of India.

The coral reef ecosystem is well known for its prodigious density and diversity of inhabitants, hence sometimes referred to as marine rain forests (Mulhall 2008). However, many species in the reef ecosystem remain overlooked due to their non-charismatic and/or cryptic nature as well as smaller size (Shima et al. 2010). Such species include one of the sessile gastropods, the vermetids. Vermetids do not have a normal coiling shell and become untwisted; in post-larval stages, the irregularly coiled shells are firmly attached to rock or any other hard substrates. These meandering tubes resemble the polychaete worm tubes (Rudiger 1995), hence the name vermetids. It is thought that there are more than 160 living species in warm temperate and tropical environments (Golding et al. 2014). The Gulf of Kachchh is situated on the western coast of India and located between 22°15'N and 23°40'N latitude and 68°20'E and 70°40'E longitude. The Gulf of Kachchh is the only site in Gujarat bestowed with one of the four major coral reef formations of the country (Singh et al. 2004). Patchy coral formations are evident on intertidal sandstones in the Gulf. The present study was carried out on Paga Reef, a submerged reef situated at the western part of the Gulf (Figure 1). The shape of the reef is oval, extending east to west with prominent fringes on northern side.

Vermetids are distributed in the marine habitats of the pan-tropical and subtropical bands around the globe (44°S to 44°N latitude) usually close to coral reefs diffusion boundaries (Safriel 1975). Present-day vermetids build dense aggregates in high-energy environments (Brachert et al. 2007). These biogenic structures are mainly consisted by *Dendropoma* species together with coralline algae, serpulids and encrusting foraminiferans (Brachert et al. 2007). The largest vermetid, *Dendropoma maximum* Sowerby, 1825, is common and widespread throughout the Indo-Paciﬁc (Hadﬁeld et al. 1972; Hughes and Lewis 1974; Zvuloni et al. 2008).

In terms of biodiversity, coral reefs are an exceptionally diverse ecosystem showcasing a large number of species interactions (Menge and Sutherland 1976). Such interactions, such as predation and competition, have often been studied in order to understand the positive and negative impacts of each organism on the others (Abrams 1987). For example, a study at Moorea revealed a negative correlation between the density of vermetids and the percentage cover of live coral (Shima et al. 2010). Furthermore, the incidence of ﬂattened coral growth forms was associated with the presence of vermetids on French Polynesia (Shima et al. 2010). The strength of these deleterious interactions argues strongly for an urgent need to improve our knowledge regarding the ecological role of vermetid gastropods as well as their interactions with corals. Therefore, the present study is aimed at describing the density, diversity and association preferences of vermetids in combination with various biotic and abiotic factors. It should be noted that the present study was focused only on field surveys for baseline data collection; it did not involve laboratory experiments or collections.

In the Gulf of Kachchh, the presence of vermetids were recorded at Adatra reef, where they form thick encrustations over hard substrata and can survive in the extreme physical conditions such as prolonged exposure due to high tidal amplitudes (Pillai et al. 1979). In addition to this, the Zoological Survey of India (2004) recorded the single vermetid species *Thylacodes variabilis* Hadfield & Kay, 1972 (as *Serpulorbis*), from various locations inside and outside the Gulf of Kachchh *i.e.*, Adatra, Okha, Porbandar, Bedi, Mithapur and Danda (ZSI 2004). However, these studies did not emphasize the density of vermetids, nor their association with live corals. The vermetids are found in association of various coral species which give rise to a number of alterations to the growth and survival of corals (Shima et al. 2010). Hence, the present study is the first of its kind to assess the density and association preferences of the vermetids on Paga reef, in the Gulf of Kachchh (Figure 1).

Paga reef has abundant reef biodiversity including both fauna and flora except for the sand patch exposed at the western end of the reef. The coral assemblages show very diverse forms on this reef, including a variety of sponges, sea anemones, zoantharians, tube worms, crabs, gastropods, bivalves and echinoderms. Altogether, they contribute more than 200 species of marine invertebrates in this locality (Singh et al. 2004). It has been observed that the density of coral decreases from the edge to the centre of the reef with some exceptional tidal pools. During the present survey, a total of 11 species of coral were recorded, the majority of which were *Favia speciosa* (Dana, 1846), *Favia favus* (Forskal, 1775), *Porites lutea* (Quoy & Gaimard, 1833), *Porites compressa* (Dana, 1846), *Turbinaria peltata* (Esper, 1794) and *Goniopora planulata* (Ehrenberg, 1834) among others.

Vermetids were evident as soft-bodied organisms with a bright orange, pale red or dark magenta colouration and covered in an upright calcareous tube, which was sometimes slightly elevated. The vermetid shells, originally dull white to earthy grey in colour, were observed to be well-encrusted by epibionts such as calcareous algae and live coral. The length of the vermetid tube emerging out of the live coral substratum was 2–3 mm and ranged from 3–8 mm in other substrates, like rock covered by silt or sand. In some places, the irregularly coiled tubes of the vermetids were also found in completely exposed conditions. The present study summarises the occurrence of three genera of vermetids *i.e.*, *Ceraesignum*, *Thylacodes* and *Petaloconchus* that showed densities of 7.7, 5.3 and 0.4 individuals/100 m^2^ respectively.

## Method

Paga reef is located between 22°28.8' to 22°30.0'N latitude and 69°11.6' to 69°15.0'E longitude covering an area of 1472.4 ha which remains submerged during high tide and gets exposed only during low tides. Therefore, the field work was carried out during a small window of low spring tides falling in daylight. The reef area was surveyed using ten belt transects measuring 100 m × 1 m in the intertidal zone (English et al. 1997). The transects were orientated perpendicular to the reef edge in order to cover a variety of substrates prevailing at reef edge and reef flat. The coordinates were recorded with an E-trex Garmin hand-held GPS navigator. The observations include survey of the vermetids along with the associated coral species or any other substratum. The study involved four major substrate types *viz.*, live coral, silt over rock, rubble, algae over rubble. One-way ANOVA was performed to evaluate the variation in vermetid densities among substrate types.

## Results

**Figure 2.**
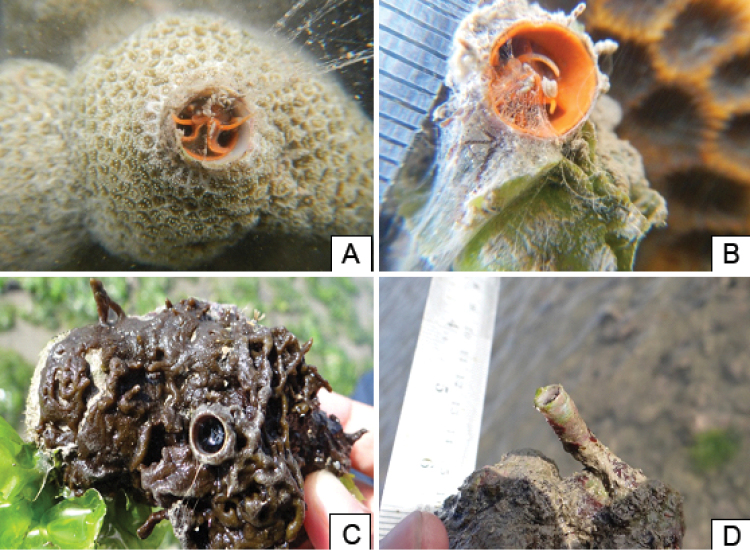
Showing association of vermetids with various substrata and epibionts **A** *Thylacodes* sp. attached with *Porites lutea* showing mucus net and pedal tentacle **B** a brightly coloured *Thylacodes* sp. with active pedal tentacles at positions 11 o’clock and 4 o’clock **C** *Ceraesignum* sp. embedded in an algae covered rubble **D** erect, uncovered *Petaloconchus* sp. tube attached to rubble.

**Figure 3.**
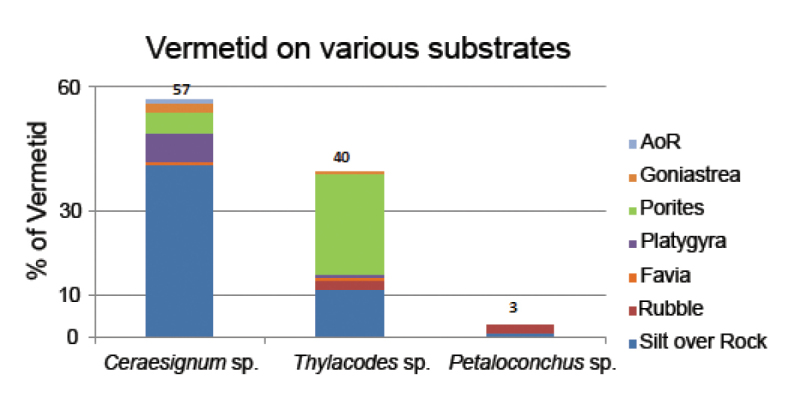
Percentage of vermetids on various reef substrates.

A total of three genera of vermetids belonging to Family Vermetidae (Subclass Caenogastropoda, Order Littorinimorpha, Superfamily Vermetoidae) was recorded from the study sites namely, *Ceraesignum* Golding, Bieler, Rawlings & Collins, 2014, *Thylacodes* Guettard, 1770 and *Petaloconchus* Lea, 1843 (Figure 2). The organisms were present in all reef zones across the intertidal belt of Paga reef but primarily concentrated on available hard substrata. Among the three genera, *Ceraesignum* spp. (7.7 individuals/100 m^2^) showed the highest density followed by *Thylacodes* spp. (5.3 individuals/100 m^2^) and *Petaloconchus* spp. (0.4 individuals/100m^2^). The generic composition in the survey is 57% of *Ceraesignum* spp., 40% *Thylacodes* spp. and 3% *Petaloconchus* spp. Furthermore, the association of each genus with the substrate type was different, illustrated in the histogram (Figure 3). *Ceraesignum* was mostly associated with silt on rock while *Thylacodes* was associated with the coral *Porites*.

Overall, the occurrence of vermetids was recorded on seven different substrates, *i.e.*, silt on rock (thin veneer of silt on rock, SoR), rubble, *Favia* Milne Edwards, 1857, *Platygyra* Ehrenberg, 1834, *Porites* Link, 1807, *Goniastrea* Milne Edwards & Haime, 1848 and algae over rubble (AoR). Maximum numbers of individuals were recorded on SoR and a minimum on AoR. However, there is no significant difference for substrate preference among the three genera (F = 1.923, df = 9.445, p = 0.1993). The species-specific preference of each vermetid species for a substrate is described below. All vermetid species were solitary: no gregarious species were found during the survey.

### *Ceraesignum* (Golding et al., 2014) (synonym *Dendropoma*)

**Figure 4.**
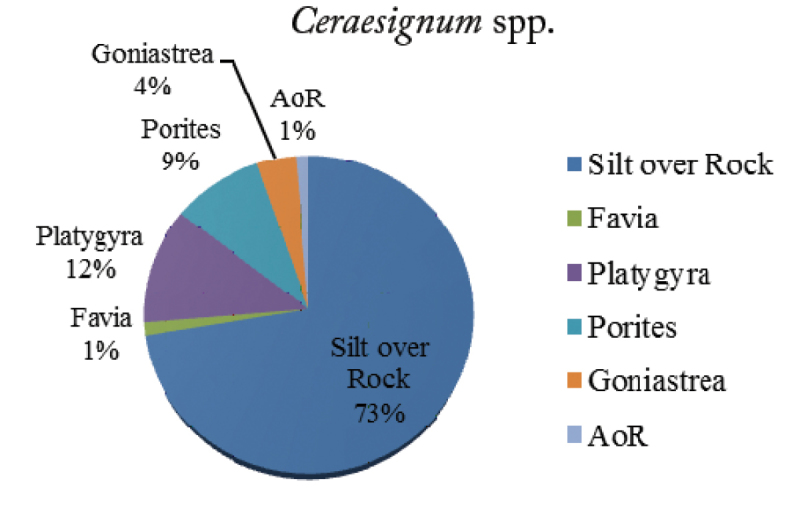
Percentage of *Ceraesignum* spp. on various reef substrates.

The density of the large vermetid *Ceraesignum* was 7.7 individuals/100 m^2^ found on six different substrates. It remained embedded in the massive and submassive coral colonies or rock, except a short part of the tube opening. The individuals of this genus showed the highest association with SoR (73%) and were recorded the least number of times on *Favia* Milne Edwards, 1857 (1%) and AoR (Figure 4). If the substrates are divided as per the biotic and abiotic categories, 73% of the total *Ceraesignum* has been recorded on abiotic substrates and 27% on live coral and algae. Moreover, among the biotic substrates, 26% of the individuals were found embedded in living coral colonies. *Ceraesignum* showed association with a total of four genera of live corals: *Platygyra* (12%), *Goniastrea* (4%), *Favia* (1%) and *Porites* (9%). It is noteworthy that three of the four coral genera belong to family Faviidae; among all the live corals, maximum individuals were found on the live colonies of *Platygyra*.

### *Thylacodes* (Guettard, 1770)

**Figure 5.**
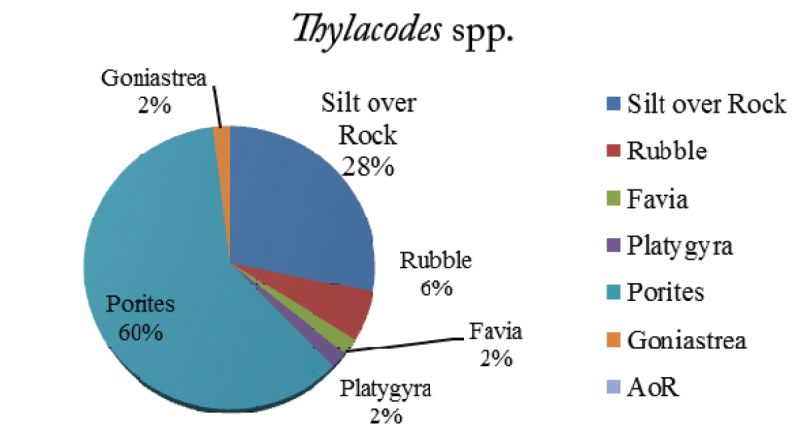
Percentage of *Thylacodes* spp. on various reef substrates.

The density of *Thylacodes* was 5.3 individuals/100 m^2^, which is less compared to *Ceraesignum* spp. The individuals of this genus were found associated with six different substrates: SoR, rubble, *Porites*, *Platygyra*, *Favia* and *Goniastrea* (Figure 5). Among these, 34% of the animals were recorded on some abiotic substrates *viz.*, SoR and rubble, and 66% of the individuals were recorded on live corals. A total of four live coral genera were recorded: *Porites* (60%), *Platygyra* (2%), *Goniastrea* (2%) and *Favia* (2%). The highest density of *Thylacodes* was recorded on *Porites* spp. It implies that more than 50% of the animals were found embedded in the live coral colonies of *Porites*. The animals were recorded without an operculum as bright orange “spots” in the dark green *Porites*. *Thylacodes* spp. shells, originally dull white to earthy grey in colour, were observed well-encrusted by epibionts such as calcareous algae and live coral. The length of the vermetid tube emerged out of the live coral substratum was 2–3 mm and ranges approximately 3–8 mm in records of presence in other substrates like rock covered by silt or sand.

### *Petaloconchus* (Lea, 1843)

**Figure 6.**
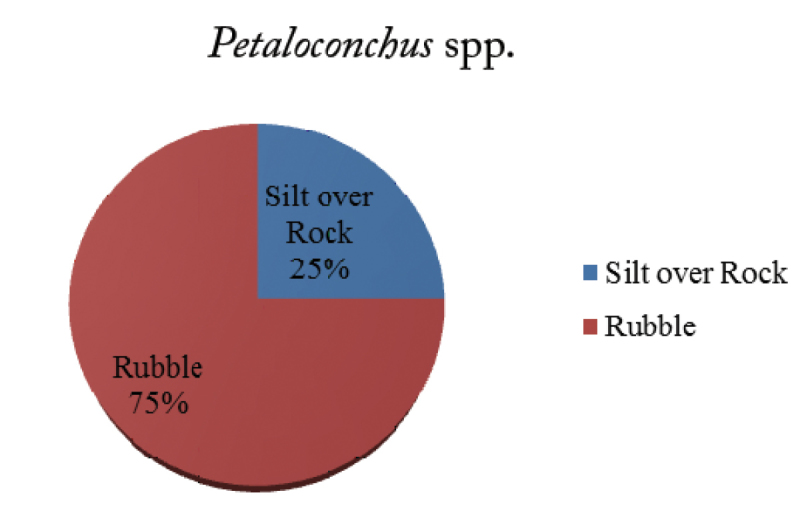
Percentage of *Petaloconchus* spp. on various reef substrates.

The genus *Petaloconchus* was represented by only four individuals and the density was 0.4 individuals/100 m^2^. The majority (75%) of the individuals were recorded on rubble and the remaining 25% on SoR (Figure 6). The animal shells were evident as erect tubes growing on the rubble and sometimes encrusted by epibionts like calcareous algae. Not a single animal was recorded on live coral colonies.

## Discussion

The present study revealed that vermetid density on Paga reef varies with the genera; however, the results of ANOVA shows that there is no significant difference in the density of the vermetids on different substrates. Hence, individuals of all the three genera are distributed evenly on seven different substrates on Paga reef. Additionally, it was observed that not a single individual of *Ceraesignum* spp. was recorded on barren rubble and no *Petaloconchus* spp. were recorded on live coral colonies. A total of 17% *Ceraesignum* spp. were observed on Faviidae corals with large corallite sizes, whereas 60% of *Thylacodes* were recorded on *Porites*, a genus with small corallite size. The association of the genus *Thylacodes* with live coral is higher than *Ceraesignum*; however, there is no significant difference (p > 0.05) between the means of both the genera.

The density of *Ceraesignum* spp. has resulted as 7.7 individuals/100 m^2^, which is much less compared to other studies worldwide. Hadﬁeld (1972) recorded the density of another species of *Ceraesignum* (as *Dendropoma gregarium* Hadfield & Kay, 1972) as high as 60,000/m^2^ on a water-levelled bench at Diamond Head, Oahu. Smalley (1984) studied the effects of various factors on population density of the solitary vermetid *Ceraesignum maxima* on Luminao Barrier Reef, Guam. These studies revealed that the vermetid density between the coral heads ranged from 0–520 individuals/m^2^ and on the *Porites lutea* coral heads it ranged from 15–520 individuals/m^2^, which are far higher than the records of the present study. This may be attributed to the large range? of substrate availability on Luminao Barrier Reef and Oahu. Additionally, Smalley (1984) recorded 78% of the population attached to the exposed sides of the coral heads whereas the present study revealed that 73% of the population attached to various type of dead substratum on reef flat.

Shima et al. (2010) recorded that the presence of vermetids was strongly associated with growth anomalies of the reef-building coral, *Porites lobata*. As well as providing the geological survey of a vermetid reef of the Salento Peninsula, Brachert et al. (2007) showed occurrences within two different settings of the Messinian reef complex: along the shallower seaward portion of the platform edge and in the upper part of the slope, in the transition zone between *Porites* colonies and bio-clastic accumulations. Smalley (1984) also studied the population density of vermetids over the coral heads of *Porites lutea*. This association preference is concordant with the present study consisting of an association of vermetids with the massive coral *Porites lutea* and additionally, the new associations of vermetids with *Favia speciosa*, *Goniastrea pectinata* and *Platygyra* sp., which has revealed novel research. Moreover, the authors have also observed a stronger association of vermetids with *Porites lutea* and *Porites compressa* on other reefs of the Gulf of Kachchh (unpublished work). Hadﬁeld (1972) also recorded the strong association of *Petaloconchus keenae* with *Porites* *i.e.*, 590 individuals/m^2^ of the *Porites* colony, whereas *Petaloconchus* spp. was recorded only on abiotic substrates in the present study.

The impact of the largest species of vermetid gastropod, *Dendropoma maximum*, on the corals revealed that the vermetid gastropod reduces skeletal growth of corals by up to 81% and coral survival by up to 52%, presumably by an unknown mechanism involving the mucus nets (Shima et al. 2010). These significant effects suggest that the interaction between vermetids and corals (regardless of the underlying mechanisms) has the potential to reduce coral cover and alter coral reef community structure (Shima et al. 2010). Hence, it is required to identify the interaction of vermetid-coral association in the GoK. Among all the living coral genera, some vermetids (*Thylacodes*) showed the highest association with *Porites* on Paga Reef. *Porites* is a dominant and frequently encountered coral genus and plays a significant role in community composition on this reef. It is frequently distributed at a variety of reef zones., starting from tidal pools at back reef areas and the reef flat to up to the reef edge with massive as well as submassive growth forms.

## Conclusions

The present work brings forth the density, diversity and habitat preferences of three vermetid genera at Paga Reef, in the Gulf of Kachchh, India. The study reveals the occurrence of vermetids on variety of substances. The organisms are distributed in all the reef zones across the intertidal belt of the reef. In spite of the adaptability of vermetids to a large range of substrates and reef zones, their density remains limited on Paga Reef compare to other reef areas worldwide. The study recommends an urgent need to identify the organisms at more detailed taxonomic levels with their respective habitat preferences.
